# Five-Year Trend in Adherence Rate to Aerobic Physical Activity Guidelines among Korean Adults in Metropolitan Cities: 2016–2020 Korea Community Health Survey

**DOI:** 10.3390/ijerph19159226

**Published:** 2022-07-28

**Authors:** Hyo Lee, Damien Vitiello

**Affiliations:** 1 Department of Sport and Health Promotion, Sangmyung University, Seoul 03016, Korea; 2 Institut des Sciences du Sport-Santé de Paris (I3SP-URP3625), Université Paris Cité, 75006 Paris, France; damien.vitiello@u-paris.fr

**Keywords:** physical activity, surveillance, COVID-19, disparities, inequity, environment, socioeconomic status

## Abstract

This study investigated the five-year (2016–2020) trend in physical activity adherence rate (PAR)—before and after the onset of the COVID-19 pandemic—and its association with socioeconomic status and community environment among Korean adults. Data were analyzed from the Korea Community Health Survey 2016–2020 concerning adults (19–65 years old) living in seven metropolitan municipalities of South Korea (*N* = 190,761). The independent variables were socioeconomic status (household income and educational attainment) and community environment (density of physical activity facilities and social cohesion), and the dependent variable was the PAR, as measured by the International Physical Activity Questionnaire-Short Form, a recall-based subjective survey. Multilevel logistic regression models with age, sex, and body mass index as covariates were applied. The results showed that the PAR increased from 37.26% (2016) to 40.84% (2019), during the pre-COVID-19 pandemic, but it sharply decreased to 31.59% (2020) during the COVID-19 confinement (trend *p* < 0.001); PAR disparities were observed following socioeconomic status, as indicated by household income (trend *p* < 0.001) and educational attainment (trend *p* < 0.001). Furthermore, significant moderation effects were observed from socioeconomic status and community environment, but the direction of the effects varied by indicator and COVID-19-related confinement period. Lower educational attainment and higher income were associated with a greater decrease in PAR during the pandemic. This study shows that PAR substantively decreased in South Korea during the COVID-19 pandemic, although it had shown a steady increase through 2019. In addition, PAR disparities by socioeconomic status were found, and socioeconomic status and community environment had distinct influences on PAR trends, depending on their indicators and pre- and post-pandemic periods. Lower educational attainment is associated with greater decreases in PAR during the pandemic, suggesting the necessity for a public campaign to maintain a physically active lifestyle during such social disaster.

## 1. Introduction

The benefits of physical activity for the prevention of noncommunicable chronic diseases have been well documented [1]. In addition, studies show that regular engagement in physical activity can boost immune function, entailing an improved prognosis for some communicable diseases, including COVID-19 [2]. Accordingly, the World Health Organization (WHO) recommends that healthy adults should engage in 150 min of moderate-intensity aerobic physical activity, 75 min of vigorous-intensity aerobic physical activity, or an equivalent amount of time of a combination of moderate- to vigorous-intensity aerobic physical activity per week [3].

However, studies show that as many as 27.5% of adults worldwide did not adhere to aerobic physical activity guidelines in 2016 [4]. In addition, the economic burden of physical inactivity is substantial. According to Ding et al. [5], global health care costs due to physical inactivity amounted to $54.8 billion international dollars in 2013. Moreover, it was reported that compliance was significantly lower in economically developed countries than in less-developed ones [4]. The physical activity adherence rate (PAR) at the national and community levels in Korea has been monitored since 1998 using population-representative surveys. A recall survey using the Global Physical Activity Questionnaire (GPAQ) found that the adherence rate was far lower than the global average, even decreasing from 58.3% to 47.8% from 2014 to 2019 [6].

A recent literature review showed a sharp decrease in physical activity and an increase in sedentary behavior worldwide since the beginning of the COVID-19 pandemic among both healthy and unhealthy (with and without cardiovascular disease) individuals [7]. However, no representative population study on the trends in PAR among South Koreans was observed between the pre- (before 2020) and post- (2020 and after) confinement periods. Therefore, how the PAR changed in South Korea before and after the COVID-19 outbreak is worth investigating.

In addition, identifying how disparities in physical activity have changed before the pandemic and during it is important. A sociological study [8] found that individuals’ participation in physical activity can be attributed to multilevel factors, ranging from intraindividual (e.g., motivation or biological propensity) to environmental (e.g., rural or urban residence) determinants. However, inequities exist in social and environmental conditions that enable or disenable a physically active lifestyle, resulting in physical activity disparities [9,10,11]. For example, an individual who resides in a physical activity-friendly environment is more likely to have a physically active lifestyle; communities that offer such an environment are likely to be economically advantaged [12]. In response to the growing problem of inequity in physical activity, the Health Plan 2030 for South Koreans has set the goals of increasing aerobic PAR to 62.8% and decreasing PAR disparities across socioeconomic classes, establishing a community-centered foundation for physical activity, and creating a physical activity-friendly environment and increased accessibility [13].

However, as noted above, restrictions on movement due to COVID-19 brought new problems such as limited access to physical activity resources and increased public concern regarding infection. These led to decreased PAR worldwide [14,15]. Related to these, studies have shown that both tangible and intangible social and environmental factors are associated with resilience during social disasters. For example, studies have shown that social cohesion is positively associated with better health behaviors, improved community health, and greater resilience to pandemics [16,17,18]. Murillo et al. [19] also reported that residents of communities with higher social cohesion are physically more active than their counterparts who have lower social cohesion despite the unfavorable community conditions such as poor walkability and low accessibility to sports facilities.

In sum, it was important and timely to investigate how the PAR changed before and after the COVID-19 outbreak and to identify any relationship between PAR trends and socioeconomic and environmental conditions. National surveillance data provides useful information about the trends in health behaviors and their correlates. The Korea Community Health Survey (KCHS) is one of South Korea’s ongoing health surveillances. Also, since it provides population-representative estimates of health behavior and health status at city, county, and district levels, the multi-level (i.e., person- and community-level) association between social and environmental conditions and physical activity participation can be tested [20].

This study, therefore, investigated the five-year trend (including the 2016–2019 period before the COVID-19 confinement period and 2020 during the COVID-19 confinement period) and disparities in physical activity adherence among Korean adults using KCHS data. This study also tested the moderation effects of personal socioeconomic status, community social cohesion, and the built environment on changes in the five-year PAR.

## 2. Materials and Methods

### 2.1. Study Design and Participants

This study analyzed data from the KCHS for 2016, 2017, 2019, and 2020; data from 2018 were omitted since the physical activity com-ponents were not surveyed in this cycle. The KCHS has been administered beginning in 2008 to produce municipality-level health statistics comparable to those available at the city, county, and district levels and to inform evidence-based health promotion programs. This survey incorporated a two-stage stratified (i.e., community and household as first and second stratum, respectively) probability proportionate sampling design [18]. The sample included in the present analyses was of noninstitutionalized adults between 19 and 65 years of age (Mean = 45.64, SD = 12.16) residing in the seven metropolitan municipalities of South Korea (*N* = 190,761). Participants who identified themselves as students were excluded from this survey since their level of education was not determined during the survey.

### 2.2. Measurements

Physical activity was measured using the International Physical Activity Questionnaire-Short Form (IPAQ-SF), which collects the frequency and duration of moderate and vigorous intensity of physical activity lasting longer than 10 min over the previous seven days. In this study, PAR was defined as the adherence rate of people who met the WHO recommendation for moderate-to vigorous intensity of aerobic physical activity.

Individual-level socioeconomic status was measured using self-reported household income (4 levels) and educational attainment (4 levels). Household income was categorized into 4 categories: <2 million Korean won (KRW) from 2 to 4 million KRW, from 4 through 6 million KRW, and >6 million KRW per month. The minimum monthly wage for full-time workers in 2022 was 1.91 million KRW (1 million KRW = 776 US dollars in 2022). The educational attainment was categorized as elementary school or below, middle school, high school, or college or above.

Community-level variables were social cohesion and the density of physical activity facilities in the built environment. Social cohesion was measured using aggregate scores for perceived mutual trust and social support among community members in the 2019 KCHS. The scores for mutual trust and social support were standardized and combined to generate a composite score for social cohesion. The density of physical activity facilities was indexed by the number of sport facilities per 1000 persons. The number of sport facilities was collected by the Statistics of Urban Planning in 2019 and made publicly available on the KCHS website (https://chs.kdca.go.kr/chs/recsRoom/dataBaseMain.do accessed on 14 June 2022). This included gyms, swimming pools, tennis courts, soccer fields, and other kinds of facilities, both public and private. On average, the number of sport facilities per 1000 persons was 0.79 with a standard deviation of 0.24.

Age, sex, marital status, body mass index (BMI), and smoking status were included in this study as demographic and behavioral covariates. Among these, BMI and smoking status were self-reported. Descriptive statistics of the participants’ characteristics are presented in Table 1.

### 2.3. Statistical Analyses

Age- and sex-adjusted population estimates of PAR across survey years, socioeconomic groups, and community attributes were calculated. Multilevel (level 1 = individual; level 2 = community) logistic regression models were used to estimate the association between PAR and survey year, socioeconomic status, and community attributes.

Model 1, in addition to the five-year trend of PAR, tested PAR disparities using the individual and community level variables of socioeconomic status and community characteristics, respectively. It included the household income, educational attainment, number of sport facilities, social cohesion, survey year, and demographic and behavioral covariates as independent variables.

Model 2 tested the moderation effects for person-level socioeconomic class regarding the five-year trend in PAR. It included the interaction terms of survey year and individual-level socioeconomic status (e.g., household income and educational attainment), in addition to the values tested in model 1.

Model 3 tested PAR disparities by community-level characteristics and moderation effects of these community attributes on the five-year trend. It included social cohesion and number of physical activity facilities and their interaction terms with the survey year, in addition to model 1.

## 3. Results

**Figure 1:**
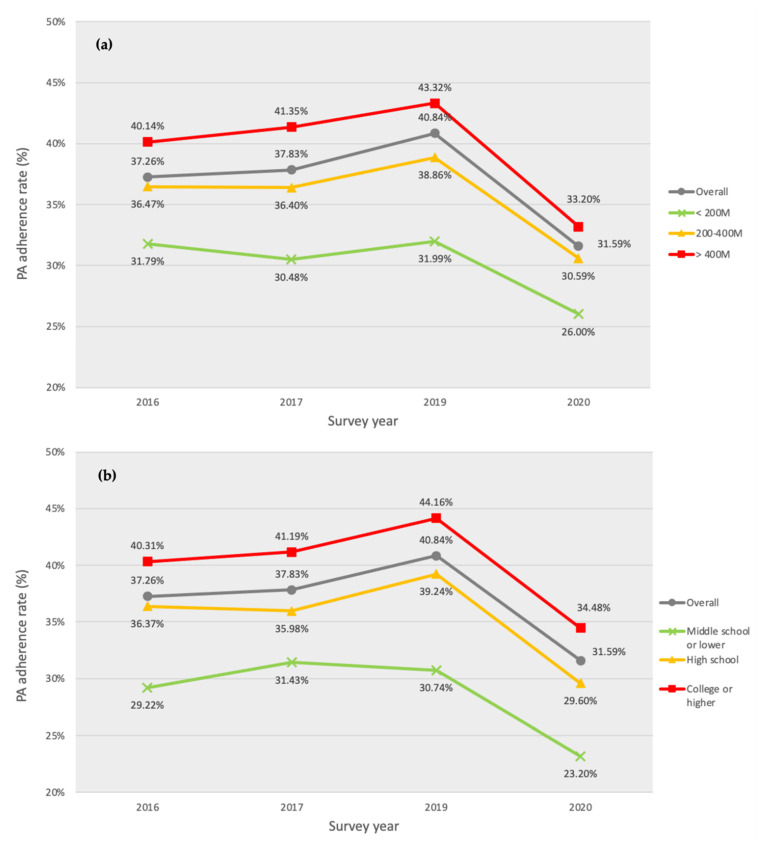
Five-year aerobic physical activity guideline adherence rate trends by socioeconomic status (**a**) household income (**b**) educational attainment (completion of the designated education level).

**Figure 2:**
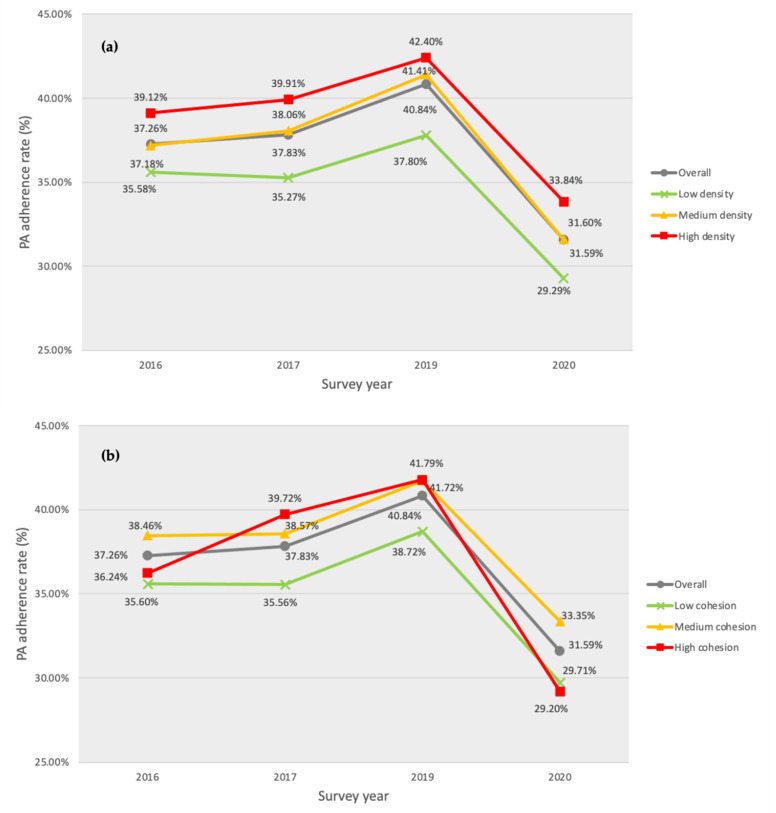
Five-year aerobic physical activity guideline adherence rate trends by community characteristics. (**a**) Density of sport facilities (number of sport facilities per 1000 persons), and (**b**) social cohesion.

Age- and sex-adjusted estimates of PAR by year over socioeconomic groups and community characteristics are presented in Figure 1 and Figure 2, and Table 2 and Table 3.

### 3.1. Model 1: Five-Year PAR Trends and Disparities by Socioeconomic Status and Community Attributes

Overall, PAR showed a significant increase from 2016 to 2019 (37.26%–40.84%; *p* for trend < 0.001), followed by a sharp decrease in 2020 (31.59%; *p* < 0.001).

The groups with the highest household income and/or college or higher diploma education were most likely to meet the PA guideline (vs. lowest income group, OR = 1.25, *p* < 0.001; vs. completion of middle school or lower education OR = 1.39, *p* < 0.001; Figure 1a,b, respectively). The number of sport facilities (OR = 1.13, *p* = 0.189; Figure 2a) and social cohesion (OR = 1.02, *p* = 0.118; Figure 2b) were not significantly associated with PAR.

### 3.2. Model 2: Moderation Effects of Socioeconomic Status on the Five-Year PAR Trend

Smaller PAR increases were observed in the lowest income group than in the highest income group in 2016–2019 (OR = 1.16, *p* = 0.001). However, in 2020, relative to 2019, the PAR decrease was smaller among those with the lowest incomes than among their counterparts with the highest incomes (OR = 1.11, *p* = 0.025).

The group with a high school diploma showed a statistically non-significant smaller PAR increase during 2016–2019 than among their counterparts with college or higher degrees (OR = 1.09, *p* = 0.063). In 2020, on the contrary, groups with middle school (OR = 0.89, *p* = 0.017) or high school education (OR = 0.92, *p* = 0.010) showed a significantly greater decrease in PAR than their counterparts with a college degree or higher educational attainment.

### 3.3. Model 3: Moderation Effects of Community Attributes on the Five-Year PAR Trend

Even though the number of sports facilities and level of social cohesion did not show significant main effects, they significantly moderated PAR increase during the 2016–2019 period. The number of sport facilities was significantly associated with greater PAR increase in 2019 (vs. 2016, OR = 0.82, *p* = 0.001). However, social cohesion was associated with lower PAR increase in 2019 (vs. 2017, OR = 1.04, *p* < 0.001). None of the community attributes were significantly associated with a decrease in PAR in 2020. Three multilevel logistic regression models described above are shown in Table 4.

## 4. Discussion

The PAR among adults in the seven metropolitan residents of South Korea showed a small but significant increase between 2016 and 2019 (before confinement due to COVID-19), but a sharp decrease in 2020 (during confinement). This study found that PAR disparities across socioeconomic status still existed. Two community-level attributes (e.g., the number of sports facilities per 1000 persons and social cohesion), meanwhile, were not significantly associated with PAR. Nonetheless, both community-level attributes and individual-level socioeconomic status significantly moderated PAR changes before and during the COVID-19 pandemic.

The PAR among Korean adults living in the seven metropolitan cities reached a peak of 40.84% in 2019 and then dropped to 31.59% in 2020. The PAR of 40.84% was far lower than the Korean national goal of 62.8% even in 2019, as indicated in the Health Plan 2030 [21].

Physical inactivity has become aggravated among both healthy and unhealthy people during the COVID-19 pandemic [7]. The decrease in physical activity during the COVID-19 pandemic may be largely attributable to the level of lockdown policy in place. For example, according to Blom et al. [22], 77% of Swedish adults reported no change in daily physical activity due to the pandemic, although 50% of Australians reported a decrease. Swedish policy during the COVID-19 pandemic has been regarded as among the softest in Western countries, while Australia maintained a hard lockdown policy beginning with the first wave of the pandemic [23,24].

Meanwhile, intranational as well as international comparisons across surveillances concerning PAR should be only with caution since the tools used for measurement differ by surveilling agents. For example, the Korea National Health (KNHANES) and Nutrition Examination Survey and the National Health and Nutrition Examination Survey (NHANES) have collected domain-specific physical activity using Global Physical Activity Questionnaire (GPAQ) in a typical week, whereas the KCHS uses the short form International Physical Activity Questionnaire, which collects nondomain-specific physical activity over the past seven days. In 2019, the PAR reported by the KHNAHES was 52.6%, 11.8% higher than the KCHS result of 40.84%. In the United States, meanwhile, the PAR re-ported by the NHANES for 2017–2018 was 68.1% [25], which was 30.3% higher than the KCHS result (37.83%) in 2017.

In this study, socioeconomic status significantly correlated with PAR, which is consistent with previous studies [8,26]. A dose–response relationship of socioeconomic status, measured by household income, and level of educational attainment with odds of adherence to aerobic physical activity recommendations were found. The mechanisms by which socioeconomic status influences physical activity could have been better understood if all domains of physical activity are measured separately. In literature reviews, for example, O’Donoghue et al. [27] and Stalsberg and Pedersen [28] reported that socioeconomic status is only positively associated with leisure-time physical activity. Individuals with low socio-economic status tend to have fewer financial resources available to participate in leisure. Furthermore, people who have low socioeconomic status are less likely to live in a walkable community and thus tend to perform less leisure-time physical activity than their counterparts with higher socioeconomic status [12]. However, studies also show that occupational physical activity is inversely associated with socioeconomic status. According to Bláfoss et al. [29], for example, workers with physically demanding jobs (i.e., members of lower social classes who are likely to engage in more occupational physical activity) tend to perform less leisure-time physical activity. Future studies should seek to measure domain-specific physical activity to better address this issue.

Community attributes indicated by the number of sports facilities and social cohesion did not show a significant main effect concerning the odds of PAR. These results are inconsistent with previous studies finding that access to physical activity facilities and higher social cohesion were positively associated with physical activity in Western countries [16,17]. Kim [30] reported that social cohesion in rural communities is positively associated with physical activity for Korean adults. Discrepancies bet period between this study and previous reports may be attributable to cultural differences (e.g., collectivistic vs. individualistic groups) or the level of urbanization (i.e., metropolis vs. rural communities). These points need to be studied in future research.

Individual-level socioeconomic status and community-level attributes, however, showed significant moderating effects on the PAR trend. First, the group with the lowest household income showed significantly smaller increases in PAR than its counterpart at the highest income level during the pre-pandemic period. Although the moderation effect did not reach statistical significance (*p* = 0.063), in addition, the group with the lowest level of education (vs. the highest level of education) showed a tendency to have smaller increases in PAR than the group with the highest education level. These imply that physical activity disparities across socioeconomic groups were magnified during this period, despite the national public health goal of reducing them [21].

The moderating effects of income level and educational attainment on PAR decline paradoxically operated in opposite directions during the COVID-19 pandemic. That is, the group with the lowest income level showed smaller decreases in PAR than its counterpart with the highest income level; simultaneously, the group with the lowest educational attainment showed a greater decrease in PAR than its counterpart, with the highest educational attainment. Explaining this phenomenon using the existing literature may be necessary despite the lack of conclusive evidence to explain this paradox due to the limited information provided by the KCHS data.

First, household income is a disposable monetary resource as well as being an addition to indicator of socioeconomic class. The ability to use paid sports facilities is proportional to disposable income. COVID-19 lockdown policies have affected physical activity all over the world, including in South Korea [31,32]. Most paid sports facilities were affected by the lockdown policy during this period, and their use was restricted during the pandemic. However, public places, public parks, which provide low- to no-cost physical activity opportunities (e.g., walking or biking), were less affected by the lockdown policy [33]. Those who had easier access to paid sports facilities before the pandemic may have needed to look elsewhere during the lockdown period and would have been likely to experience a greater decrease in physical activity than their counterparts with a lower income level.

Second, quality education can provide knowledge and skills for a physically active lifestyle [34,35]. According to theories of social psychology, appropriate knowledge, attitudes, and perceptions of one’s competence are antecedents of participation in physical activity [36,37]. For example, individuals who recognize the importance of physical activity for their health and wellbeing (i.e., knowledge and attitude) and have an appropriate level of physical activity literacy (i.e., skills and competence) would have been more likely to have maintained their level of physical activity during the COVID-19 pandemic. This suggests that a need for public campaigns and/or lifelong education on the importance and practical knowledge of the physical activity, especially during social disasters, was noted.

Between 2016 and 2019, larger numbers of sports facilities in the community tended to be accompanied by larger increases in PAR despite a nonsignificant main effect. This is logically consistent with a significant and positive moderating effect on household income during the same period (i.e., before the COVID-19 lockdown, people with higher incomes took greater advantage of sports facilities than their counterparts with lower incomes). The number of sports facilities, nevertheless, did not significantly moderate PAR changes during the COVID-19 pandemic. This seems to be attributable to the lockdown policy due to its limited use.

However, higher social cohesion was associated with smaller in-creases in PAR between 2017 and 2019. Nevertheless, one should use caution in concluding that social cohesion had a negative impact on physical activity. That is due to the fact that individuals living in a community with higher social cohesion tended to be more physically active although the difference was not significant (*p* = 0.118). Moreover, higher social cohesion was positively associated with greater increases in PAR in 2016–2017 (OR = 1.02, *p* = 0.002; these statistics were obtained by changing the reference year to 2016; see also (Figure 2b). This implies that a ceiling effect for the group with higher social cohesion may be seen for 2017–2019 since a greater increase took place during the 2016–2017 period. If this is interpreted positively, the inverse interaction effect implies that physical activity disparities across communities with higher and lower social capital decreased between 2017 and 2019.

It is believed that this is the first study to examine the association between PAR trends and socioenvironmental factors across the pre-pandemic and during the pandemic of COVID-19. A particular strength of this study was that the participants were randomly selected and represented the population of each metropolitan municipality in South Korea. However, this study also has some limitations. First, physical activity was measured using the IPAQ-SF, a self-report subjective survey, which is by its nature susceptible to recall and social desirability bias. Second, social cohesion and the number of sports facilities were measured in 2017 and has not been updated since, so the variables were assumed to be invariant over time. Readers should also recognize that the kinds of social and environmental factors that affect physical activity may not be limited to social cohesion and the number of sports facilities. These factors, according to the social ecological model of physical activity participation [38], encompass various components of the intra-individual (e.g., motivation, perceived barriers and benefits, and self-efficacy) to public health policy aspects of society. Future studies should address these issues by incorporating objective measures of physical activity and more comprehensive social–ecological perspectives.

## 5. Conclusions

This study tested the five-year trend in PAR and its associations with socioeconomic status and socioenvironmental characteristics of community in Korean adults living in seven metropolitan municipalities. Overall, individuals with higher socioeconomic status were more likely to adhere to physical activity guidelines. PAR values increased from 2016 to 2019 but substantively decreased under the COVID-19 lockdown (2020). Moderation analyses showed that the effects of socioeconomic status and social–environmental characteristics had distinct influences on PAR trends before and after the outbreak of COVID-19. The greater decrease in PAR was associated with lower educational attainment during the pandemic period. This underlines the importance of massive dissemination of knowledge concerning the benefits of adopting active behavior at school but outside as well, especially during a social disaster, such as a communicable disease pandemic.

## Figures and Tables

**Table 1:** Description of study participants.

Participant Characteristics	Categories	*n*	%
Age group	20s	24,127	12.55
	30s	39,252	20.41
	40s	47,647	24.78
	50s	51,708	26.89
	60s	29,543	15.36
Sex	Male	86,420	44.95
	Female	105,857	55.05
Marital status	Married	130,115	67.76
	Divorced/widowed/separated	19,456	10.13
	Never married	42,442	22.1
BMI	<18.5	8371	4.38
	18.5 to <25	127,297	66.57
	25 to <30	48,081	25.14
	30 or higher	7487	3.92
Smoking	Not smoking	152,129	79.13
	Smoking	40,129	20.87
Household income	<200 M KRW	29,779	15.49
	200–400 M KRW	66,763	34.72
	400–600 M KRW	52,742	27.43
	>600 M KRW	42,993	22.36
Educational attainment ^1^	Middle school or lower	26,746	13.93
	High school	68,980	35.92
	College or higher	96,314	50.15

^1^ Completion of the designated education level.

**Table 2:** Five-year aerobic physical activity adherence rate by socioeconomic status (age- and sex-adjusted).

	Household Income ^1^	PAR (%)	95% CI%		Educational Attainment ^2^	PAR (%)	95% CI%	
2016	Overall	37.26%	36.77%	37.74%	Overall	37.26%	36.77%	37.74%
	<200	31.79%	30.62%	32.95%	Middle school or lower	29.22%	26.73%	31.71%
	200–400	36.47%	35.69%	37.25%	High school	36.37%	35.54%	37.21%
	>400	40.14%	39.34%	40.95%	College or higher	40.31%	39.43%	41.20%
2017	Overall	37.83%	37.34%	38.32%	Overall	37.83%	37.34%	38.32%
	<200	30.48%	29.21%	31.75%	Middle school or lower	31.43%	28.76%	34.11%
	200–400	36.40%	35.60%	37.21%	High school	35.98%	35.13%	36.82%
	>400	41.35%	40.59%	42.11%	College or higher	41.19%	40.35%	42.02%
2019	Overall	40.84%	40.32%	41.37%	Overall	40.84%	40.32%	41.37%
	<200	31.99%	30.32%	33.67%	Middle school or lower	30.74%	27.72%	33.77%
	200–400	38.86%	37.89%	39.83%	High school	39.24%	38.32%	40.16%
	>400	43.32%	42.61%	44.02%	College or higher	44.16%	43.31%	45.01%
2020	Overall	31.59%	31.11%	32.07%	Overall	31.59%	31.11%	32.07%
	<200	26.00%	24.63%	27.36%	Middle school or lower	23.20%	20.57%	25.83%
	200–400	30.59%	29.74%	31.44%	College or higher	29.60%	28.78%	30.42%
	>400	33.20%	32.54%	33.85%	College	34.48%	33.73%	35.23%

^1^ Currency amounts are in million Korean won (KRW = about 756 US dollars in 2022). ^2^ Completion of the designated education level.

**Table 3:** Five-year aerobic physical activity adherence rate by community attributes (age- and sex-adjusted).

	Density of Sport Facilities ^1^	PAR (%)	95% CI%		Social Cohesion ^2^	PAR (%)	95% CI%	
2016	Overall	37.26%	36.77%	37.74%	Overall	37.26%	36.77%	37.74%
	Low	35.58%	34.59%	36.56%	Low	35.60%	34.69%	36.51%
	Medium	37.18%	36.52%	37.85%	Medium	38.46%	37.77%	39.14%
	High	39.12%	38.10%	40.15%	High	36.24%	35.23%	37.25%
2017	Overall	37.83%	37.34%	38.32%	Overall	37.83%	37.34%	38.32%
	Low	35.27%	34.30%	36.24%	Low	35.56%	34.64%	36.49%
	Medium	38.06%	37.38%	38.74%	Medium	38.57%	37.89%	39.25%
	High	39.91%	38.90%	40.93%	High	39.72%	38.69%	40.74%
2019	Overall	40.84%	40.32%	41.37%	Overall	40.84%	40.32%	41.37%
	Low	37.80%	36.71%	38.88%	Low	38.72%	37.73%	39.71%
	Medium	41.41%	40.69%	42.13%	Medium	41.72%	40.99%	42.46%
	High	42.40%	41.34%	43.47%	High	41.79%	40.69%	42.88%
2020	Overall	31.59%	31.11%	32.07%	Overall	31.59%	31.11%	32.07%
	Low	29.29%	28.32%	30.25%	Low	29.71%	28.82%	30.60%
	Medium	31.60%	30.94%	32.25%	Medium	33.35%	32.68%	34.03%
	High	33.84%	32.85%	34.82%	High	29.20%	28.24%	30.17%

^1,2^ Low, medium, and high groups represent the first, second, and third tertiary groups, respectively.

**Table 4:** Multilevel logistic regression analyses models of demographic characteristics, socioeconomic status, community characteristics, and year associated with aerobic physical activity adherence.

Independent Variables	Model 1 (Year + Covariates)				Model 2 (Model 1 + Year × SES Interaction)				Model 3 (Model 1 + Year × Community Attributes Interaction)			
	Coeff	OR	95% CI	*p* Value	Coeff	OR	95% CI	*p* Value	Coeff	OR	95% CI	*p* Value
Age	0.004	1.00	1.00–1.01	<0.001	0.004	1.00	1–1.01	<0.001	0.004	1.00	1.00–1.01	<0.001
Sex	−0.579	0.56	0.55–0.57	<0.001	−0.579	0.56	0.55–0.57	<0.001	−0.579	0.56	0.55–0.57	<0.001
Marital status	Reference = never married				Reference = never married				Reference = never married			
Divorced/widowed/separated	−0.008	0.99	0.96–1.03	0.651	−0.006	0.99	0.96–1.03	0.722	−0.007	0.99	0.96–1.03	0.699
Married	0.163	1.18	1.14–1.21	<0.001	0.163	1.18	1.14–1.21	<0.001	0.163	1.18	1.14–1.21	<0.001
BMI	0.008	1.01	1.01–1.01	<0.001	0.008	1.01	1.01–1.01	<0.001	0.008	1.01	1.01–1.01	<0.001
Smoking	Reference = not smoking				Reference = not smoking				Reference = not smoking			
Yes	−0.168	0.85	0.82–0.87	<0.001	−0.168	0.85	0.82–0.87	<0.001	−0.168	0.85	0.82–0.87	<0.001
Household income ^1^	Reference = low income				−0.402	0.67	0.62–0.72	<0.001	Reference = low income			
Medium	0.225	1.25	1.21–1.29	<0.001	−0.106	0.9	0.86–0.94	<0.001	0.226	1.25	1.21–1.29	<0.001
High	0.331	1.39	1.35–1.44	<0.001	Reference = high income ^5^				0.332	1.39	1.35–1.44	<0.001
Educational attainment ^2^	Reference = middle school or lower				−0.326	0.72	0.68–0.77	<0.001	Reference = middle school or lower			
High school	0.235	1.27	1.22–1.31	<0.001	−0.084	0.92	0.88–0.96	<0.001	0.236	1.27	1.22–1.31	<0.001
College or higher	0.329	1.39	1.34–1.44	<0.001	Reference = college or higher ^6^				0.330	1.39	1.34–1.44	<0.001
Density of port facilities ^3^	0.121	1.13	0.94–1.35	0.189	−0.402	1.13	0.94–1.35	0.187	0.163	1.18	0.97–1.43	0.096
Social cohesion ^4^	0.020	1.02	0.99–1.05	0.118	−0.106	1.02	0.99–1.05	0.118	0.009	1.01	0.98–1.04	0.537
Year	Reference = 2019				Reference = 2019				Reference = 2019			
2016	−0.094	0.91	0.89–0.94	<0.001	−0.133	0.88	0.84–0.91	<0.001	0.094	0.91	0.89–0.94	<0.001
2017	−0.087	0.92	0.89–0.94	<0.001	−0.085	0.92	0.88–0.96	<0.001	−0.088	0.92	0.89–0.94	<0.001
2020	−0.393	0.67	0.66–0.69	<0.001	−0.371	0.69	0.66–0.72	<0.001	−0.394	0.67	0.66–0.69	<0.001
Year × household income					Reference = 2019 × highest income							
2016 × lowest					0.144	1.16	1.06–1.26	0.001				
2016 × medium					0.032	1.03	0.97–1.1	0.306				
2017 × lowest					0.009	1.01	0.92–1.1	0.839				
2017 × medium					−0.046	0.96	0.9–1.01	0.139				
2020 × lowest					0.106	1.11	1.01–1.22	0.025				
2020 × medium					0.021	1.02	0.96–1.09	0.517				
Year × education					Reference = 2019 × college or higher							
2016 × middle school or lower					−0.001	1.00	0.91–1.09	0.982				
2016 × high school					0.020	1.02	0.96–1.08	0.511				
2017 × middle school or lower					0.084	1.09	1.00–1.19	0.063				
2017 × high school					0.015	1.02	0.96–1.08	0.612				
2020 × middle school					−0.116	0.89	0.81–0.98	0.017				
2020 × high school					−0.079	0.92	0.87–0.98	0.010				
Year × sport facilities									Reference = 2019			
2016 × density of sport facilities									−0.183	0.83	0.75–0.93	0.001
2017 × density of sport facilities									−0.058	0.94	0.85–1.05	0.297
2020 × density of sport facilities									0.082	1.09	0.97–1.21	0.153
Year × social cohesion									Reference = 2019			
2016 × social cohesion									0.010	1.01	0.99–1.03	0.207
2017 × social cohesion									0.035	1.04	1.02–1.05	<0.001
2020 × social cohesion									−0.001	1.00	0.98–1.02	0.895
Intercept	−0.521	0.59	0.49–0.72	<0.001	0.144	1.15	0.96–1.39	0.124		0.65	0.57–0.74	<0.001

*N* = 190,761 ^1^ Monthly household income categories: lowest ≤2 million KRW, lower= 2 million to <4 million KRW, higher = 4 million to <6 million KRW, highest = 6 million KRW or higher. ^2^ Completion of the designated education level. ^3^ Number of sport facilities (swimming pools, health clubs, gyms, and so on) per 1000 residents. ^4^ Sum of the standardized scores on mutual trust and social support items, aggregated to the community-level. ^5,6^ Reference groups were changed to avoid multicollinearity problems when interaction terms with categorical variables are introduced into the model.

## Data Availability

Data used in this study are available upon request at https://chs.kdca.go.kr/chs/stats/statsMain.do accessed on 14 June 2022.
